# Single-Cell RNA Analysis of Type I Spiral Ganglion Neurons Reveals a *Lmx1a* Population in the Cochlea

**DOI:** 10.3389/fnmol.2020.00083

**Published:** 2020-05-25

**Authors:** Fiorella Carla Grandi, Lara De Tomasi, Mirna Mustapha

**Affiliations:** ^1^Cancer Biology Program, Stanford University, Stanford, CA, United States; ^2^Department of Biomedical Science, University of Sheffield, Sheffield, United Kingdom; ^3^Department of Otolaryngology-Head and Neck Surgery, Stanford University School of Medicine, Stanford, CA, United States

**Keywords:** type I spiral ganglion neurons, single-cell transcriptome, *Lmx1a*, development, cochlea

## Abstract

In the mature cochlea, each inner hair cell (IHC) is innervated by multiple spiral ganglion neurons of type I (SGNI). SGNIs are morphologically and electro-physiologically diverse. Also, they differ in their susceptibility to noise insult. However, the molecular underpinnings of their identity and physiological differences remain poorly understood. In this study, we developed a novel triple transgenic mouse, which enabled the isolation of pure populations of SGNIs and the analysis of a 96-gene panel *via* single-cell qPCR. We found three distinct populations of Type I SGNs, which were marked by their exclusive expression of *Lmx1a, Slc4a4*, or *Mfap4/Fzd2*, respectively, at postnatal days P3, P8, and P12. Our data suggest that afferent SGN subtypes are established genetically before the onset of hearing and that the expression of key physiological markers, such as ion channels, is heterogeneous and may be underlying the heterogeneous firing proprieties of SGNIs.

## Introduction

The inner and outer hair cells (IHC and OHC) of the organ of Corti allow us to perceive sound using spiral ganglion neurons (SGNs). SGNs type I and type II, which innervate the IHC and OHC, respectively, are structurally and functionally different. Type I SGNs (SGNI) make up 90–95% of SGNs and are the main cells that transmit complex sound information to the brain (Berglund and Ryugo, 1987; Nayagam et al., 2011). The remaining 5–10% of type II SGNs (SGNII) are the sensory drive for the olivocochlear efferent reflex (Froud et al., 2015).

In the mature cochlea, each IHC is innervated by multiple SGNI fibers that have varying morphological and electrophysiological properties, such as low and high thresholds of sound detection. Heterogeneous electrophysiological features of postnatal SGNI can be distinguished tonotopically and within the same cochlear region (Davis and Liu, 2011). In addition to their functional and morphological differences, studies have suggested that SGNIs also exhibit differential vulnerability to aging and noise-induced excitotoxicity. Single-fiber recording studies have shown a selective reduction of high-threshold neurons in noise-exposed and aging rodents, resulting in auditory synaptopathy (Kujawa and Liberman, 2015) The mechanisms, as to why high-threshold fibers are predominantly affected by noise remain elusive.

SGNs and vestibular ganglion neurons (VGNs), which are both derived from the proneurosensory tissue, are defined early in development by the transcription factor networks of GATA3 and TLX3, respectively (Appler and Goodrich, 2011). However, little is known about the subsequent mechanisms involved in specifying the terminal differentiation of SGNIs to low- and high-threshold neurons. The identities of other molecular selectors, be they intrinsic or extrinsic to the cell, remain unknown. Recent studies of the retina may provide clues about the developmental regime operating in the inner ear. Lineage tracing studies of retinal progenitor cells have determined that they are multipotent and they differentiate *via* defined, irreversible states (Goetz et al., 2014). Although these progenitors can, to some degree, be influenced by extrinsic cues, a growing list of transcription factors have been suggested as intrinsic regulators of retinal cell specification. Many of these genes also affect hearing, leading us to hypothesize that SGNI subtypes are also genetically defined by intrinsic cues.

Validating this hypothesis requires the ability to specifically sort out and profile single SGNIs from cochlear tissue. With this goal, we established a transgenic mouse model capable of differentially fluorescently labeling SGNI and SGNII. This allowed us to isolate pure, single-cell populations and perform single-cell transcriptomic analysis. The single-cell transcriptomic analysis is a powerful tool to understand cellular diversity in complex tissues, and has been successfully used in the inner ear (Durruthy-Durruthy et al., 2014; Waldhaus et al., 2015; Petitpré et al., 2018; Shrestha et al., 2018; Sun et al., 2018). However, these previous studies focused primarily on adult SGNs. To test our hypothesis about the intrinsic genetic definition of SGN subtypes before the onset of hearing, we profiled SGNs at postnatal day 3 (P3) and P8, before the onset of hearing and at P12, around the onset of hearing in most mice. Using a 96-gene targeted single-cell RT-PCR platform, we identified and validate three main clusters of SGNIs in the neonatal ear. *Lmx1a*, *Slc4a4*, and *Mfap4/Fzd2* designate the three clusters, respectively. This targeted approach allowed us to amplify low-abundance genes that were absent from other studies.

## Materials and Methods

### A Mouse Model for SGN Labeling

All the animal experiments were performed following institutional and governmental regulations approved by the Stanford University Institutional Animal Care and Use Committee. A triple transgenic mouse line was generated by systematically crossing three lines: Ai14-tdTomato (Jax:007908) mice were crossed with Bhlhb5-cre mice, a neuronal-specific transcriptional factor (Lu et al., 2011). These mice were subsequently crossed with peripherin (*Prph*)-GFP mice (McLenachan et al., 2008; Huang et al., 2014) to generate triple transgenic *Ai14*-tdTomato, *Bhlhb5*-cre, *Prph*-GFP mice. Peripherin is a type III intermediate filament protein expressed in SGNIIs (Hafidi et al., 1993). In this scheme, SGNI cells are labeled in red, and SGNII is labeled in red and green. We have used a similar approach to create the *Lmx1a* reporter line. We have crossed a *Lmx1a*-cre (Chizhikov et al., 2010) to *Ai14*-tdTomato and *Prph*-GFP mouse line.

### Cochlea Dissociation and Cell Culture

For single-cell experiments, 4–6 of each of the postnatal ages P3, P8 or P12 cochleae were incubated in digestion solution [50 μM kynurenic acid (Sigma–Aldrich, K3375), 10 mM MgCl_2_, 10 mM glucose in MEM Hanks (Life Technologies, 11575-032)] with 50 μg/ml collagenase (Roche, 10269638001) and 6 μg/ml DNAseI (Worthington, LS002004) for 15 min at 37°C with continuous shaking at 50 rpm (Excella E24 Incubator Shaker Series, New Brunswick Scientific). Tissue was dissociated with gentle pipetting four to six times during digestion. Subsequently, trypsin (Gibco, 15090046) was added to a final concentration of 0.05%, and tissues were and incubate for another 15 min at 37°C and 50 rpm. For P12 cochleae, we replaced trypsin with milder recombinant enzyme 0.05% TrypLE Select (Gibco, A12177-01) for better cell viability. After digestion, the cell suspension was placed on ice and remaining clumps were dissociated by pipetting. The enzymatic digestion was stopped using the fetal bovine serum. The samples were centrifuged at 0.8× *g* for 5 min at 4°C, and cells were resuspended in 500 μl HBSS (Hyclone, ADD20159) and passed through a 35 μm cell strainer (Corning, 352235) and used directly for fluorescence-activated cell sorting (FACS) analysis or culture.

To prepare neuronal cultures, the cells were resuspended in Neurobasal-A media supplemented with glutamax (Gibco, 35050079), 1× B27 (Gibco, 17504-044), 10 ng/ml BDNF (Sigma, B3795) and 10 ng/ml NT-3 (Sigma, N1905), and cultured overnight on 0.5 mg/ml poly-D-lysine (Sigma, P6407) coated coverslip in a 35 mm cell culture dish.

### Immunostaining and Neuron Quantification

Cells cultured overnight were fixed with 4% paraformaldehyde in PBS for 30 min at room temperature, then were washed three times for 10 min in room temperature PBS. Cells were blocked with 5% BSA/0.5% Triton-X 100/PBS for 1 h at room temperature, then washed three times in PBS. Cells were incubated overnight with the TUJ1 antibody (BioLegend, 801202) at a 1:500 dilution at 4°C, then washed three times with 0.1% Tween20 in PBS for 10 min at room temperature, before incubating with secondary antibody for 1 h and repeating wash steps. Slides were mounted with anti-fade mounting media with DAPI (Invitrogen, 1010789). Cells were manually counted from different areas on coverslip under a 20× fluorescent microscope.

### Single Neuron Sorting by Fluorescence-Activated Cell Sorting (FACS)

Cochleae were dissected out from triple transgenic animals and enzymatically dissociated as described in the cochlea dissociation section. Cells were then stained with Sytox red (Life Technologies, S34859) and sorted on the FACS Aida and FACS Falstaff (BD Biosciences) at the Stanford FACS core facility. Cells debris and dead cells were removed by gating forward scatter area (FSC-A) and side scatter area (SSC-A; Supplementary Figures S1D–H for FACS gating strategies). Finally, tdTomato and GFP positive cells were gated and high tomato positive cells were sorted into 96-wells plate, with each well, containing 5 μl of 2× reaction mix (Invitrogen, CellsDirect, 1753-500) mixed with 0.05 units of SUPERase-In RNase inhibitor (Ambion, PN AM2696) and stored at −80°C until use. The total time from animal sacrifice to single-cell sorting was ~2.5 h. A subset of cells was always kept for culture to ensure minimal cell stress to the cells being analyzed by single-neuron qRT-PCR.

### Single-Neuron Multiplex qRT-PCR

Single-neuron multiplex qRT-PCR assays were performed on sorted cells following the manufacturer’s guidelines (Fluidigm manual-PN 68000088 L) and as previously described (Durruthy-Durruthy et al., 2014). Briefly, each cell was placed in a well containing CellsDirect reagents (Invitrogen, CellsDirect, 11753-500) to isolate RNA. RNA was then transcribed to cDNA, and specific target genes were pre-amplified with one step PCR using SuperScript III RT Platinum Taq Mix and 500 nM primer (DELTAgene). Samples were treated with ExoI (NEB, M0293L) to cleave off single-stranded DNA. Exo-treated samples were diluted five times with nuclease-free water. Samples were then prepared for qRT-PCR analysis as per manufacturer specifications (Fluidigm, 85000736). qRT-PCR experiments were performed on the Biomark HD (Fluidigm manual, PN 68000088 L1) with pre-defined protocol GE96.96 Fast PCR+Melt v2.PCI for 30 cycles using the 96.96 dynamic arrays integrated fluidic circuit chip (IFC, Fluidigm). Data are available in Supplementary Table S1. For P3, the data represent 203 single cells aggregated from three independent runs. For P8, the data represent 383 single cells aggregated from seven independent runs, and for P12, data represents 230 single cells aggregated from three runs. Each run consists of four to six pups pooled from a litter.

### Data Processing and Bioinformatics

A series of preliminary experiments were conducted to validate that: (a) the primers amplify single amplicons in the expected size range; (b) the target mRNA is indeed expressed in neonatal and young cochlea; and (c) to determine the limit of detection (LOD), which is the cycle threshold (Ct value) for each primer/gene combination. Quantitative single-cell RT-PCR cannot be normalized to a single housekeeping gene or groups of genes, but rather to the individually determined detection limit for each primer pair. Ultimately, quantitative gene expression for each primer pair and cell is presented as expression level above detection limit on a log scale using Log2Ex values [Log2Ex = Ct(LOD) − Ct(measured); Durruthy-Durruthy et al., 2014]. In simple terms, Log2Ex for a gene represents transcript level above background in log base 2. LOD Ct values for each primer pair were determined in dilutions of bulk cochlear cDNA (neonatal and P21) over 16 orders of magnitude. Primers that did not meet the three above stated validation requirements were eliminated. A list of 96 genes (Supplementary Table S1) was used for gene expression profiling, and the Log2Ex values were used for downstream analysis.

Before clustering, the data set was cleaned by removing cells with low expression of housekeeping genes *Gapdh* and *Actb*, as well as removing any non-neuronal contaminants by selecting cells, which expressed *Map2* and *Tubb3*. We used HDBSCAN, implemented in python, to cluster single cells obtained from P3, P8, and P12 cochlea, and obtained six clusters. In selecting our clustering method, we sought to find a method that would agree with our underlying assumptions about the cell populations, namely that: (1) the variance between different cell populations might not be the same; (2) the size of each subpopulation may be different; (3) some rare cell populations might not be sufficiently sampled; and (4) variance in the data can be introduced by additional factors, such as RNA degradation. HDBSCAN (McInnes et al., 2017) has many advantages over traditional k-means clustering, including its ability to deal with data with variable density and variance, fulfilling goals (1) and (2), and the ability to deal with noisy data by assigning some points to no cluster, fulfilling conditions (3) and (4). We restricted our analysis to five of these six clusters, as the sixth was found not to express any of the selected genes. To visualize the cells, we utilized UMAP, a dimensionality-reducing algorithm, to project the cells into 2D space and mapped the HDBSCAN called cluster identities. Here, we present all cells, including those cells that HDBSCAN did not cluster due to low confidence about their identity. Enrichment for a particular marker gene was tested using a one-factor ANOVA with correction for multiple hypothesis testing (alpha = 0.05/96), and then each significant ANOVA was tested using the *post hoc* Tukey test. Statistical analysis was performed in Python and Prism (Graphpad).

We also repeated the same clustering process using PCA analysis plus K-means clustering and hierarchical clustering. K-means clustering was applied in each data sets using the algorithm “Hartigan-Wong” with one thousand iterations (iter.max = 1,000) in R. Hierarchical clustering was computed by Ward’s minimum variance method (Ward.D2). The numbers of stable clusters generated were assessed by gap statistic (Tibshirani et al., 2001).

### RNAscope *in situ* Hybridization and Conventional *in situ* Hybridization

RNAscope *in situ* hybridization was performed according to RNAscope guidelines (ACD, document number, 320293; Wang et al., 2012). Briefly, temporal bones were removed in ice-cold PBS and cleaned. Cochleae were placed in 4% PFA at 4°C for 22–24 h with gentle shaking. After fixation, samples were washed 2× with PBS and dehydrated overnight with 30% sucrose at 4°C, for 24 h, the mounted in OCT. Fourteen micrometer cochlear section was cut using a cryostat. The manufacturer designed probes were used for double fluorescent labeling (*Cacna1a*: 493141-C2, *Mfap4*: 421391, *Kcnd2*: 452581-C3) according to manufacturer specifications. Conventional *in situ* hybridization procedures for *Cacna1a* and *Nefm* were performed as described (Mendus et al., 2014). Briefly, *in situ* probes were cloned into the pGEM-T vector (Promega, Madison, WI, USA), with the following primers: *Cacna1a*: GAGAGAATTCGGGCGCACTGCAAATGATAA and GAGAAAGCTTGTCCCAAGCCCACGTTTTTC. *In situ* hybridization was carried out as previously described (Schwander et al., 2007).

### Confocal Imaging and Signal Quantification

Images were acquired on a confocal microscope (Zeiss, LMS700) as previously described (Mendus et al., 2014). A 0.5 μm z-stack of images was collected. The signals for specific genes such as *Cacna1a*, *Mfap4*, and *Kcnd2* in each cell were visualized in Velocity 3D image analysis software (PerkinElmer, Inc., Waltham, MA, USA). The boundary of a cell was defined by merging fluorescent images with bright-field images and manually tracing cell borders. The numbers of fluorescent signals in each defined boundary were quantified manually.

## Results

### Isolation of Type I and Type II SGNs From the Cochlea

**Figure 1:**
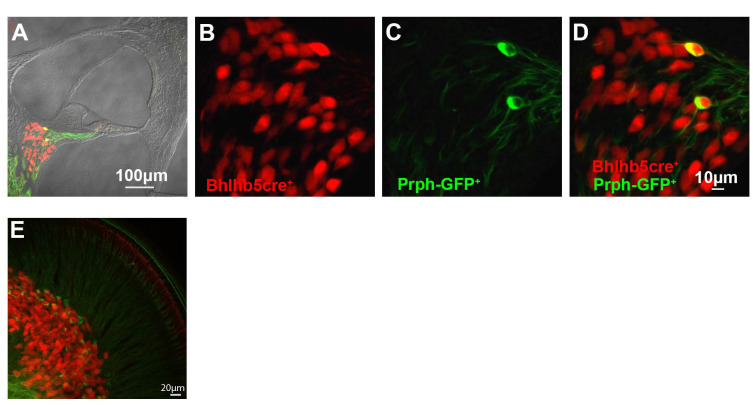
Transgenic mouse model for spiral ganglion neuron (SGN) Type I and Type II Isolation. **(A)** Cross-section of the organ of Corti showing SGNs showing tdTomato; bhlhb5Cre (SGNIs) and Prph-GFP (SGNIIs) from cryopreserved tissue. **(B,C)** Fluorescent imaging of tdTomato and GFP. **(D)** Merge of panels **(B,C)**. **(E)** Whole-mount of the organ of Corti showing cell body and projections of SGN type I (red) and type II (green) from cryopreserved tissues. All images are representative fields from experiments repeated three times (*n* = 3).

To prepare single-cell suspensions of SGNs from the cochlea, we utilized a triple transgenic mouse model in which Type I and II SGNs are uniquely labeled with different fluorescent reporters. Briefly, we crossed *Ai14*-tdTomato to *Bhlhb5*-cre mice and *Prph*-GFP mice (see “Materials and Methods” section). This resulted in cochlear tissues where SGNIs are labeled red and SGNIIs are marked red and green (Figures 1A–E). We then isolated pure cell populations by FACS for subsequent single-cell transcriptome analysis. To validate our dissociation and sorting strategy, we immunostained cells with the neuronal marker TUJ1 (Supplementary Figures S1A,B). Our dissociation protocol resulted in 85.6% cell viability after sorting (Supplementary Figure S1C). As expected, tdTomato/GFP double-positive cells (SGNIIs) compose only 0.05% of the final viable fraction (Supplementary Figures S1D–H). Cells were directly sorted into 96-well plates and analyzed using the Fluidigm single-cell platform (Supplementary Figures S2A–F). The panel of 96 genes (Supplementary Table S1) was preselected using microarray expression profiles generated from the same mouse model and contained genes hypothesized to be either selectors or effectors for SGNI subpopulations.

After quality control and filtering (see “Materials and Methods” section), the single-cell data was visualized using the UMAP projection, which visualizes high-dimensional data on a 2D axis and whose utility for single-cell data has been recently shown (McInnes et al., 2018; Becht et al., 2019). We analyzed 203 cells at P3, 383 cells at P8, and 230 cells at P12.

### *Zic1*, *Pax6*, and *Nfix* Serve as Novel Markers of Type I SGNs

**Figure 2:**
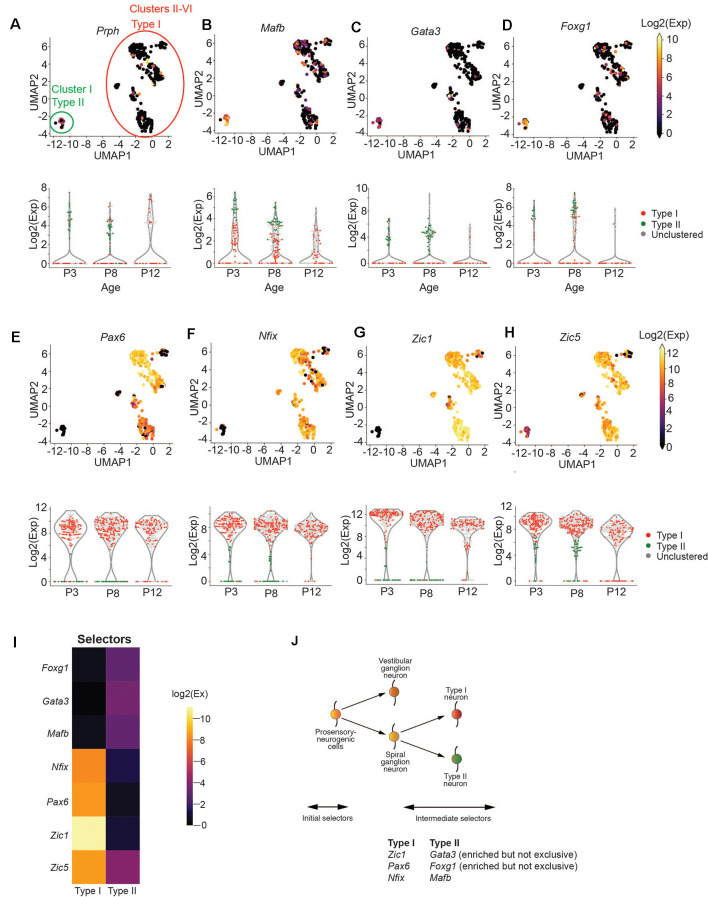
*Zic1, Zic5, Pax6*, and *Nfix* are Type I SGN markers. **(A–D)** UMAP projection of all SGNs from postnatal day 8 cochlea (*n* = 383 cells, pooled from 7 technical runs representing 28 animals). Each point represents a single cell. Type I and Type II cells are circled in red and green, respectively. Cells are colored by the expression of *Prph* **(A)**, *Mafb* **(B)**, *Gata3* **(C)**, and *Foxg1* **(D)**. The expression scale is given on the right. Violin plots for each gene are given below the UMAP plot. Each point represents a cell. Cells are colored either red (Type I), green (Type II), or gray (unclustered). **(E–H)** UMAP projections and violin plots as in (a) for *Pax6* **(E)**, *Nfix* **(F)**, *Zic1* **(G)**, and *Zic5* **(H)**. **(I)** Heatmap for effector genes. **(J)** Proposed schematic for Type I and Type II specification based on data above.

We first sought to determine the broad molecular features separating Type I and Type II SGNs at postnatal day 8 (P8), before the onset of hearing. Mapping the FACs gating information onto the UMAP projection of SGNs, we observed that Cluster I is enriched for tdTomato/GFP expressing cells, suggesting that this cluster corresponds to Type II SGNs (Supplementary Figure S3A). Classically, SGNII has been defined by the expression of *Prph* (Hafidi et al., 1993). Cells in Cluster I were enriched for *Prph* expression (Figure 2A) as well as *Mafb* and *Gata3* (Figures 2B,C), which also have been suggested as markers of postnatal Type II cells (Petitpré et al., 2018; Shrestha et al., 2018; Sun et al., 2018). We also found *Gata3*/*Mfab* positive cells that are not *Prph* positive (Figures 2A–C), as has been observed by other studies (Petitpré et al., 2018; Shrestha et al., 2018; Sun et al., 2018). The ambiguity of this expression highlights the need for better molecular markers to distinguish between Type I and Type II SGNs.

With this goal, we focused on the transcription factors in our panel to find those that might act as selectors to designate Type II vs. Type I cells. We observed that Type I cells were strongly enriched for *Pax6, Nfix*, and *Zic1* (Figures 2E–G), while Type II cells were enriched for *Foxg1* (Figure 2D). In contrast*, Zic5*, although highly expressed in Type I cells, was also mildly expressed by Type II cells (Figure 2H). We observed that while *Zic1* expression was highest in early development (P3) and decreased slightly with time, *Pax6* and *Nfix* expression stayed constant from P3 to P12 (Figures 2E–H), suggesting they would be optimal markers to identify Type I and II cells over time. In addition to these transcription factors, we find that *Cadps2* and *Tmem178* are broadly enriched in Type I SGNs (Supplementary Figure S3B), as well as *Cacna1g*, which although expressed in Type II SGNs, is more strongly expressed in Type I cells (Supplementary Figure S3B) in contrast to previous findings. Collectively, these data provide us with novel genetic markers for Type I and II SGNs (Figures 2I,J).

### Type I SGNs Cluster Into Three Major Subtypes

**Figure 3:**
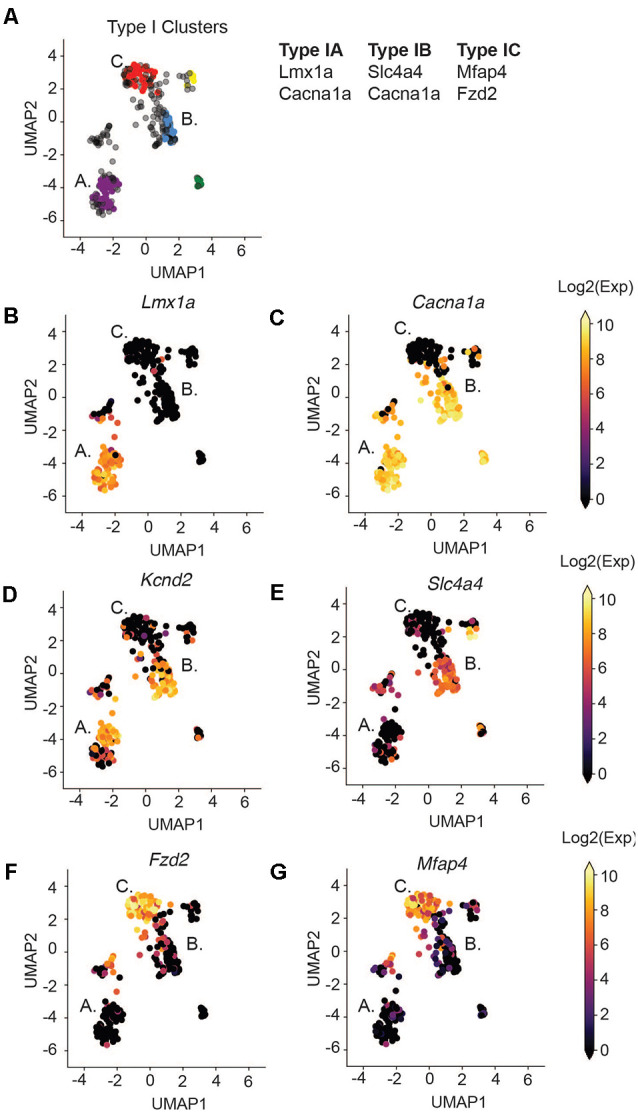
Type I SGNs cluster into three subtypes. **(A)** UMAP projection of the Type SGNs at postnatal day 8. Each point represents a cell. Cells clustering to Type IA, IB, IC are colored in purple, blue, and red, respectively. The major gene markers for each subtype are given on the right. Two additional clusters were found (in yellow and green) but are not considered in this analysis as they did not have clear marker genes. Cells in gray were not clustered into any of the subgroups. **(B–G)** UMAP projection of Type I cells. Each cell is colored by the expression of **(B)** *Lmx1a*, **(C)** *Cacna1a*, **(D)** *Kcnd2*, **(E)** *Slc4a4*, **(F)** *Fzd2*, **(G)** *Mfap4*. The expression scale is given on the right.

We next focused our attention on identifying subsets of Type I SGNs at post-natal day 8 (P8). Previous work has shown a variety of physiologically distinct SGNI cells (Taberner and Liberman, 2005; Davis and Liu, 2011) and we hypothesize that these cells should have unique molecular signatures. Therefore, we used machine learning to cluster all the Type I SGNs based on their 96-dimensional gene expression profiles. We chose to use HDBSCAN clustering because it can deal with data of variable density and variance (see “Materials and Methods” section). After data transformation, we selected cells that expressed the housekeeping genes *B-actin* and *Gapdh*, as well as high levels of neuronal markers *Map2* and *Tubb3* (Supplementary Figures S3C–F). HDBSCAN was run on this set of Type I SGNs and provided five high confidence clusters (II-VI), of varying sizes (Supplementary Figures S3G–I). Three of these clusters contained a substantial number of cells with clear delineating markers, which we termed Type IA, IB, and IC (Figure 3A). Similar delineations were found using k-means clustering following PCA analysis (Supplementary Figure S4) as well as hierarchical clustering (Supplementary Figure S5), validating our findings. Type IA, B, and C cells were distinguished by their expression of the *Lmx1a* (Figure 3B), *Slc4a4* (Figure 3E), and *Mfap4 and Fzd2* (Figures 3F,G), respectively. Both Type IA and IB cells express *Cacna1a* (Figure 3C) and *Kcnd2* (Figure 3D). The additional two subtypes were characterized by a complex pattern of gene expression (Supplementary Figure S6). For completeness, we projected all cells, including those that were not assigned to any subset, onto the UMAP axes when displaying gene expression.

Some of these marker genes have been previously implicated in SGN biology, in particular *Lmx1a* and *Cacna1a. Lmx1a* belongs to the family of LIM-domain containing transcription factors (Rétaux and Bachy, 2002) and is known to play roles in regulating fate decisions, and defining neural boundaries and domains in both the central and peripheral nervous system and the inner ear (Millonig et al., 2000; Chizhikov and Millen, 2004; Nichols et al., 2008; Koo et al., 2009). *Cacna1a* (encoding CAV2.1) has been shown to control fast excitatory synaptic transmission and low threshold exocytosis in the CNS (Jun et al., 1999; Pagani et al., 2004; Weiss and Zamponi, 2013), and its expression in SGNs has been determined by whole-cell and single-channel recordings (Lv et al., 2012, 2014; Stephani et al., 2019). Less is known about *Slc4a4* in SGNs, however, mutations in the human *SLC4A4* gene have been associated with neurosensory disorders including glaucoma and hereditary sensory neuropathy type I (Kok et al., 2003; Dinour et al., 2004). Although the *Fzd2* gene has not been characterized in SGNs, its ligand WNT5A plays an important role in planar cell polarity and cochlear development (Qian et al., 2007; Munnamalai and Fekete, 2013). Therefore, our identified marker genes are instrumental to distinguish functional subsets of SGN cells.

### The Abundance of IA, IB and IC SGNIs Changes During Neonatal Development

**Figure 4:**
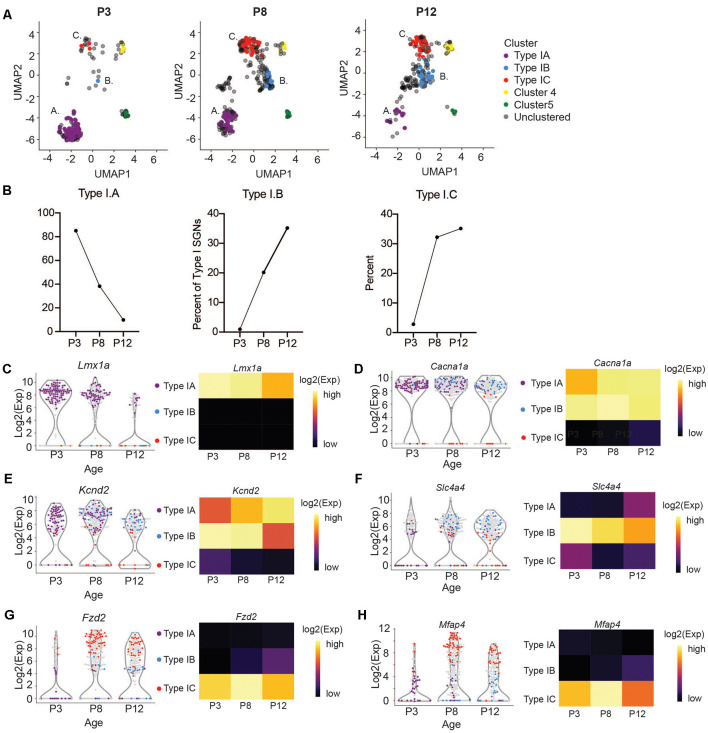
Subtypes of Type I change in abundance at the onset of hearing. **(A)** UMAP projections of Type I SGNs colored by subtypes at postnatal days 3, 8, and 12 (*n* = 203 single cells at P3 and *n* = 230 single cells at P12, representing three independent technical runs, each with four to six animals). Postnatal day 8 is reproduced from Figure 3A for clarity. **(B)** Quantification of the percentage of Type IA, IB, and IC cells as a fraction of the total Type I SGNs at each age. **(C–H)** Violin plot of **(C)** *Lmx1a*, **(D)** *Cacna1a*, **(E)** *Kcnd2*, **(F)** *Slc4a4*, **(G)** *Fzd2*, **(H)** *Mfap4*. Each point represents a single cell, colored according to its subtype or gray if outside the IA, IB, IC. On the right, a heatmap of the same data. The expression scale is giving on the right side.

We next sought to trace the number and fate of these subpopulations over-development by performing single-cell qPCR on SGNIs, before and at the onset of hearing from P3 and P12 cochlea, respectively. We clustered these cells together with P8 neurons and projected them onto the same UMAP embedding. At P3 and P12, we can see cells belonging to all the clusters we described (Figure 4A). However, we observed age-related changes in their abundance. IA cells are abundant at pre-hearing stages (P3, P8) representing 85% and 38% of the total SGNs analyzed, and decrease after the onset of hearing to 9% (Figure 4B). On the other hand, IB and IC cells are low at P3 (0.9% and 2.8%) and increase significantly at P8 (20% and 32%), stabilizing by P12 (35% and 35%; Figure 4B). The expression markers genes for the IB and IC subtypes stayed fairly constant from P8 to P12, after the large increase in these cell populations (Figures 4D–H). In contrast, expression levels of *Lmx1a* also decreased mildly with the concurrent loss of IA cells (Figure 4C). The loss of the IA population may either reflect differentiation or death with development or increased sensitivity of these cells to manipulation with age. Intriguingly, *Lmx1a* expression was not found by single-cell SGN studies focusing on the adult cochlea (Petitpré et al., 2018; Shrestha et al., 2018; Sun et al., 2018).

### SGNI Subtypes Have Distinct Physiological and Signaling Properties

We next sought to understand the unique functions or properties of the Type I SGN subtypes by analyzing the expression patterns of the other genes beyond those defining the subtypes in our assay. As demonstrated by our PCA analysis (Supplementary Figure S4D), none of these genes has a role in exclusively defining a particular subtype, but several are enriched in only one or two of the subtypes. Genes were classified by their broad functions: *transcription factor*, *signaling, physiology, guidance, and adhesion* (Supplementary Figure S6). Overall, we observed that transcription factor expression (beside *Lmx1a* and *Zeb1*) from our selected gene set was generally homogenous between all three identified subsets of Type I SGNs, while some of the signaling and physiology related genes have more distinct expression patterns among the subtypes.

**Figure 5:**
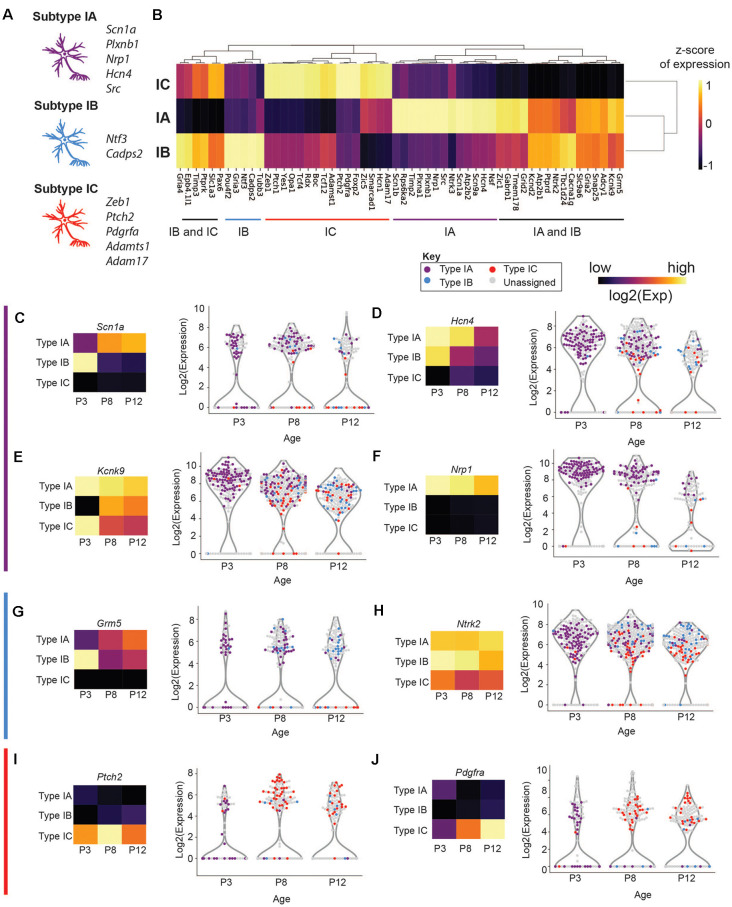
Expression of physiologically relevant genes in SGN Type I subtypes. **(A,B)** Hierarchical clustering of genes that are differentially enriched in Type I **(A,B,C)** SGNs and schematic of the most important effectors (on the left). **(C–J)** Violin plot of **(C)** *Scn1a*, **(D)** *Hcn4*, **(E)** *Kcnk9*, **(F)** *Nrp1*, **(G)** *Grm5*, **(H)** *Ntrk2*, **(I)** *Ptch2*, and **(J)** *Pdgfra*. Each point represents a single cell, colored according to its subtype or gray if outside the IA, IB, IC. On the left, a heatmap of the same data. Each square has the average expression of cells in that subtype. The expression scale and color key are on the top of the page.

We found that subtype IA cells were enriched for a variety of sodium channels, including *Scn1a* (Nav1.1) *Scn2b* (Nav1.5), and *Scn9a* (Nav1.7; Figures 5A,G, Supplementary Figure S6). These channels activate at a more negative membrane potential than the other Nav channels and therefore may contribute to making fibers sensitive to the lower intensity of sound (Royeck et al., 2008; Fryatt et al., 2009; Browne et al., 2017). In addition to these channels, the IA subtype differentially express channels that mediate SGNI resting membrane potential and control neuronal excitability such as hyperpolarization-activated cyclic nucleotide-gated channel α-subunit 2 and 4 (HCN2 and HCN4; Figures 5A,C) and the K+-selective leak channels (KCNK9; Figure 5E; Welker and Woolsey, 1974; Mo and Davis, 1997; Kim and Holt, 2013; Liu et al., 2014).

IA cells were also enriched for markers related to neural branching and patterning, suggesting that these cells may still be migrating or differentiating into their mature forms between P3 and P12. In IA cells, we find enrichment for *Plxna1* and *Plxnb1* (Figure 5A). *Plxna1*, the receptor for class 3 semaphorin (*Sema3a*) was recently shown to be involved in SGNI branching and refinement during postnatal synapse maturation (Katayama et al., 2013; Jung et al., 2019). *Plxnb1* also plays a role in axonal guidance through *Sema2a* in the CNS (Ayoob et al., 2006). Finally, IA cells are enriched in *Nrp1* (Figures 5A,F), a receptor involved in neural pathfinding, survival, and maintenance (Cariboni et al., 2011; Guaiquil et al., 2014). Together these genes expressed by the IA subtype, in addition to *Lmx1a*, maybe representing a subset of mid or low threshold neurons that are refining their final connections with target cells.

Subtype IB was enriched in physiological markers involved in increased neural excitability such as *Kcnd2* (Kv4.2; Figures 4E, 5A), a potassium voltage-gated channel. Interestingly, Kv4.2 expression in the SGNs is regulated by neurotrophins (Adamson et al., 2002). This channel is activated at membrane potentials that are below the threshold for action potentials (Shibata et al., 2000; Chen et al., 2006; Granados-Fuentes et al., 2012). Kv4.2 functions downstream of the metabotropic glutamate receptor GRM5 and plays a role in nociception mediated by activation of GRM5 (Hu et al., 2007). *Grm5* is enriched in both type IA and IB as compare to IC (Figures 5A,G). IB cells are also enriched for *Gria4* at P8 (Figure 5A), another glutamate receptor known to control the frequency, amplitude, and kinetics of the spontaneous excitatory postsynaptic channels of the reticular thalamic nucleus (nRT) neurons (Paz et al., 2011, p. 4). Taken together with the highest expression of *Cacna1* in type IB SGNI, this subtype may be a representative of the mid to low-threshold SGNI.

Additionally, subtype IB cells had the highest expression of Ca^2+^-dependent activator protein for secretion 2 (*Cadps2*; Figure 5A), which is involved in cell survival and the activity-dependent release of the brain-derived neurotrophic factor (BDNF; Sadakata et al., 2004; Shinoda et al., 2011). BDNF is involved in neuronal maturation and synaptic plasticity (Sadakata et al., 2007, 2013). Intriguingly, *Bdnf*, and *Ntf3* expression, as well as their receptor *Ntrk2*, are high in type IB, although present in the other subtypes (Figure 5A, Supplementary Figure S7). Previous studies have shown a graded expression of neurotrophins in the cochlea, with BDNF expression being highest at the basal turn, while NTF3 is highest at the apical edge (Adamson et al., 2002; Schimmang et al., 2003), suggesting that type IB cells originate from many tonotopic areas. Although expression analysis cannot definitively establish electrophysiological properties, these patterns suggest that IA and IB are more closely related molecularly than IC and may the low or mid-low neurons.

Subtype IC cells are defined by their specific expression of adhesion and signaling molecules *Mfap4* and *Fzd2*. They are also enriched for two members of the Tcf transcription factor family: *Zeb1* (*Tcf8*) and*Tcf12* (Figure 5A), which may be involved in establishing the IC cluster during early development. Several members of the hedgehog-signaling pathway, *Ptch1*, *Ptch2*, and *Yes1*, are also enriched in this subtype (Figure 5A). *Ptch2* expression (Figure 5I) has previously been shown to influence neural cell fate decisions and regulate synaptic plasticity and neuronal activity in the CNS (Konířová et al., 2017, p. 2; Herholt et al., 2018). Type IC cells also expressed high platelet-derived growth factor receptor alpha (*Pdgrfa*; Figure 5J). PDGF receptors and their ligands play essential roles in neuronal differentiation during embryonic stages and in adult neuronal maintenance (Funa and Sasahara, 2014). PDGF receptors were elevated in cochlear tissues, including SGNs, following noise injury (Fetoni et al., 2014; Bas et al., 2015), suggesting their role in cochlear tissue protection following noise trauma. These expression patterns indicate that IC neurons may be the high-threshold SGNIs. Collectively, our findings suggest that the three SGNI subtypes defined by their distinctive transcriptional profiles represent physiologically distinct subpopulations.

### Validation of Type I SGNs in the Mouse Cochlea

**Figure 6:**
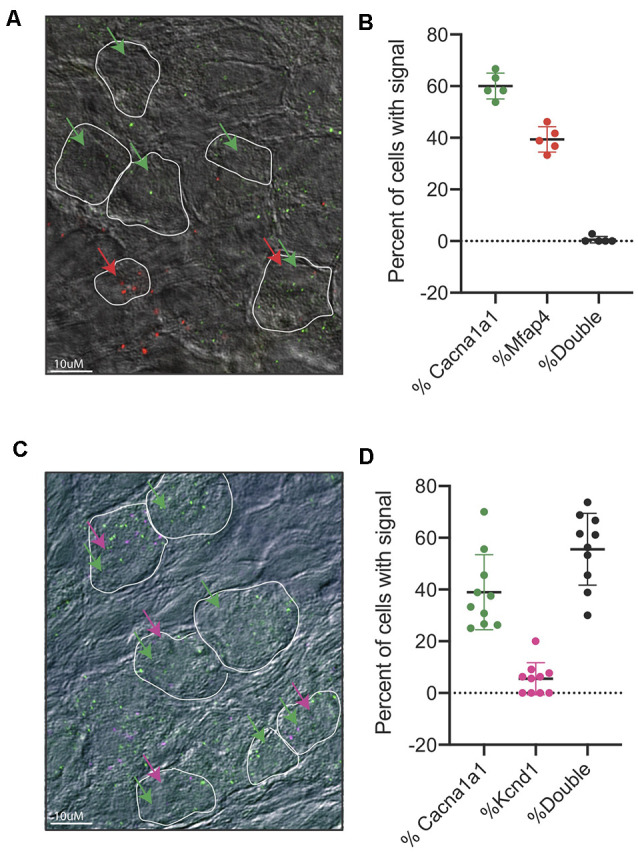
RNA-scope of *Cacna1a* and *Kcnd2*. **(A)** Representative image from RNAscope *in situ* hybridizations of *Cacn1a1* (green) and *Mfap4* (red) with bright field merge at postnatal day 8 from cryopreserved cochlear tissue. White boundaries indicate cells; green arrows indicate cells expressing *Cacna1a*, and red arrows indicate cells expressing *Mfap4*. **(B)** Quantification of cells expressing either *Cacna1* only (60% ± 5%) or *Mfap4* only (39.4% ± 4.8%) or both (0.6% ±1.25%) as percentage of all cells scored per image. Cells were considered positive if they had >1 dot per cell, 5 independent images (*n* = 5 mice) were scored. Each point represents a scored image; all data presented as average ± STD. **(C)** Representative image from RNAscope *in situ* hybridizations of *Cacn1a1* (green) and *Kcnd2* (purple) with bright field merge at postnatal day 8. White boundaries indicate cells; green arrows indicate cells expressing *Cacna1a*, and purple arrows indicate cells expressing *Kcnd2*. **(D)** Quantification of cells expressing either *Cacna1* only (38% ± 14.5%) or *Kcnd2* only (5% ± 6%) or both (55.9% ±13.9%) as a percentage of all cells scored per image. Cells were considered positive if they had >1 dot per cell, 10 independent images (*n* = 10 mice) were scored. Each point represents a scored image; all data presented as average ± STD.

After identifying IA, IB, and IC to be the main three subpopulations arising from our single-cell dataset, we sought to validate these three populations *in vivo*. First, we designed *in situ* probes against *Cacna1a*, which we observed to be enriched in both the IA and IB subtypes. Indeed, we see a strong signal of *Cacna1a* in cochlear tissues at both P3 and P8 (Supplementary Figures S6D,E). However, due to the low resolution of traditional *in situ*, we were not able to localize the expression of more than one gene to individual populations of neurons. Therefore, we opted to use RNA-scope technology, an *in situ* hybridization approach with low background signal, that allows us to visualize and/or co-localize two or more probes (Wang et al., 2012). We designed and tested probes against several of the defining population markers (see “Materials and Methods” section). For the validated probes, we were able to observe subpopulations of SGNs that exclusively expressed *Cacna1a*, representing Type IA and Type IB cells and *Mfap4* expressing cells, representing Type IC cells (Figures 6A,B). We also tested the co-localization of *Cacna1a* and *Kcnd2*. From the single-cell data, we expected to observe two populations, one that would only express the *Cacna1a* representing Type IA cells without *Kcnd2* (Figure 4G) and those expressing *Cacna1a* and *Kcnd2*, pooled both from Type IA and Type IB. In concordance with this data, in RNA-scope images of the cochlea, we observed both cells only expressing *Cacna1a* and others expressing both *Cacna1a* and *Kcnd2* (Figures 6C,D).

**Figure 7:**
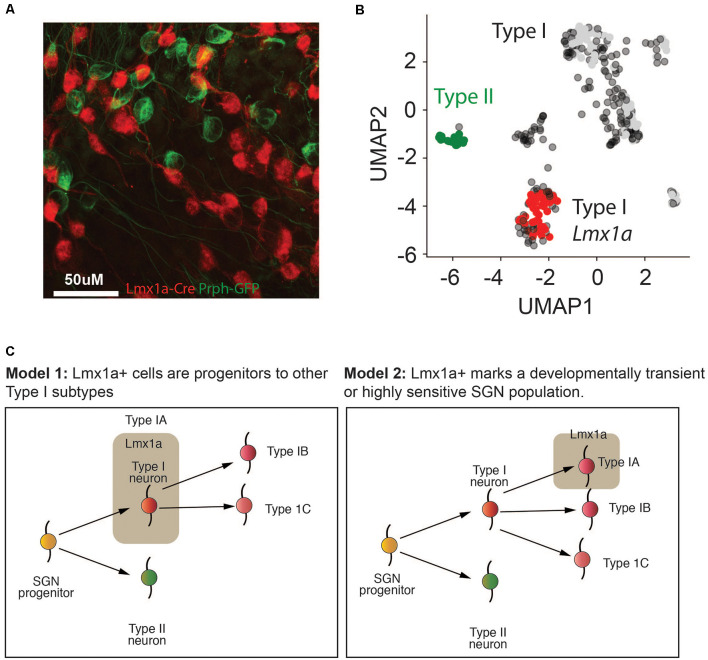
*Lmx1a* marks a distinct population of Type I SGNs.** (A)** Representative fluorescent image of *Lmx1a*-tdTomato x GFP-Prph at P8 from cryopreserved tissue. Magnification at 66×. **(B)** UMAP of P8 SGN Type I and Type II cells, colored according to the mouse model in **(A)**. **(C)** Two putative models for how the different Type I SGN subtypes identified in this article might form in the cochlea. In model 1, the Type IA *Lmx1a* cells form an early progenitor population that is more immature and gives rise to the Type IB and Type IC cells, thereby explaining the age-related decline in these cells. In model 2, the Type IA *Lmx1a* cells are an independent subtype, but they are either more sensitive to stress at later ages, because they are more sensitive, or they are a developmentally transient population that begins to decline by P8.

Previous studies had established a role for *Lmx1a* in inner ear cell fate decisions (Nichols et al., 2008; Koo et al., 2009). However, we were unable to establish robust probes for *Lmx1a*. Therefore, to validate the existence of SGNI cells which expressed *Lmx1a*, we established a new triple transgenic mouse line using a *Lmx1a*-cre line (Chizhikov et al., 2010) crossed to the *Ai14*-tdTomato reporter line and *Peripherin*-GFP lines. Our *in vivo* data revealed that only a subset of SGNIs (i.e., cells not showing GFP expression) was red at P8 (Figure 7A), validating that IA cells are indeed a unique subset of SGNIs distinct from the SGNII (Figure 7B). Further studies need to be performed on this mouse model to analyze the dynamics of this cell population over time (Figure 7C).

### Comparison to a Previously Published Dataset of P3 SGNs

We next sought to compare our study to the previously published results from whole-genome single-cell studies (Petitpré et al., 2018; Shrestha et al., 2018; Sun et al., 2018). Of these, two focused primarily on adult SGNs, and therefore were not suitable for comparison with our study, as neuronal properties are known to change from neonate to adult (Crozier and Davis, 2014). We thus chose to compare our work to the *Pvalb-cre* P3 neurons sequenced by Petitpré et al. (2018) although the two studies use different transgenic mouse models for sorting.

In both the adult and P3 data, Petitpré et al. (2018) found three subtypes of SGNs, characterized by their expression of *Calb1, Runx1, and Calb2* (Type IA), *Lypd1, Grm8 and Runx1* (Type IB) and, *Trim54, Pcdh20, Rxrg and Calb2* (Type IC). We did not have any of these markers in our targeted panel, except *Calb1*, whose expression we compared (Supplementary Figures S7A,B). We see a more limited expression of *Calb1*, although it is enriched in Type IA (*Lmx1a* positive) neurons at P3, suggesting that these two subtypes may be similar (Supplementary Figure S7B). However, by P8, we observe the majority of the *Calb1* expressing cells to be Type II cells (Supplementary Figure S7C).

We next wanted to determine if we could sort out our subtypes using the Petitpré et al.’s (2018) data. Thus, we selected the same 96 genes found in our panel, and projected and clustered the cells as before, but were not able to form the same clusters. We noted several differences between our data and Petitpré et al. (2018): (1) SGNI markers *Zic1* and *Zic5* were not expressed (Supplementary Figures S7D,E), although they are expressed in previous studies of bulk SGN tissues at P0 and P6 (Supplementary Figure S7F, Lu et al., 2011). (2) Two of our marker genes (*Lmx1a* and *Mfap4*) were not expressed (Supplementary Figures S7G,H). *Fzd2* was lowly expressed by a few of their Type IC cells, but these did not cluster together in our analysis (Supplementary Figure S7F). Finally, *Slc4a4* and *Cacna1a* were broadly expressed across all subtypes (Supplementary Figures S7J,K).

These discrepancies in the two datasets may suggest that transcripts like *Lmx1a* and *Mfap4* may be low-abundance and therefore need to be pre-amplified to be detected. The limited detection of low-abundance transcripts is a known problem with both the 10× and Smart-seq2 pipelines, as these transcripts are not efficiently converted into cDNA. Thus, our dataset complements previous whole sequencing studies that set the outlines of SGN subtypes with the detection of these low abundance transcripts.

## Discussion

In the mature cochlea, each IHC is innervated by multiple spiral ganglion neuron subtype-I (SGNI) cells that are morphologically and physiologically diverse. SGNIs possess complex endogenous firing properties that enable them to rapidly and faithfully transmit the wide dynamic range of sound information to the auditory brainstem (Taberner and Liberman, 2005; Davis and Liu, 2011). However, the factors that define the molecular and physiological diversity of SGNIs remain poorly understood. In this study, we identified marker genes that distinctively label different neonatal SGNI neuron subtypes by using a novel transgenic reporter mouse. This model was designed to distinguish the SGNI and II by their already known exclusive markers and allowed us to isolate pure populations of SGNI (*Bhlhb5*-cre × tdTomato) and SGNII (*Peripherin*-GFP) neurons by FACS. This step facilitated the construction of single-cell transcriptomes of SGNIs and SGNIIs neurons.

Using our single-cell dataset of SGNI cells from P3, P8 and P12 cochlea’s we identified three distinct populations: Type IA, marked by *Lmx1a* expression, Type IB marked by *Slc4a4* and Type IC marked by *Mfap4/Fzd2*. Although, we observed all three subtypes of Type I SGNs at each developmental age, their relative sizes varied throughout development. This might be due to the differentiation of progenitor-like SGNs at earlier time points, or selective loss of certain subtypes that are more sensitive to stress (Figure 7C). Previous work has highlighted the existence of three electrophysiological categories whose proportions changed between early postnatal (P0-P3) and adult (Crozier and Davis, 2014). Intriguingly, although a previous study also found three subtypes of SGNs at postnatal day 3 (P3; Petitpré et al., 2018), we found little overlap between the marker genes defining the populations. In particular, *Lmx1a* expression was absent from this dataset. This discrepancy between the datasets may be due to the qPCR-based technique allowing pre-amplification of low abundance transcripts, the technique of cochlear dissociation, or the choice of transgenic mouse models. These differences, however, highlight the importance of using several orthogonal methods to investigate complex biological systems, as each technique can reveal unique and complementary features of the tissue.

Previous studies have established that type I SGNs have a variety of physiological phenotypes, corresponding to the so-called low, mid and high threshold neurons (Adamson et al., 2002; Taberner and Liberman, 2005; Liu et al., 2014; Kujawa and Liberman, 2015). We hypothesized that these identities were genetically encoded early in the development of the cochlea, before the onset of hearing. Our observation that we can sort out at least three distinct subsets of neurons at P3, based on 96-gene markers, is in line with this hypothesis. To characterize the physiological identity of these subtypes, we analyzed the expression of effectors that could define and maintain a particular SGNI subtype’s electrophysiological characteristics. We found both heterogeneities between SGNI subtypes (Figure 5) and also variable expression between SGNI cells of the same subtype.

A recent modeling study of spinal dorsal horn neurons showed that within a single neuron population, varying the densities of just two ion channels could reproduce five patterns of neuronal firing (Balachandar and Prescott, 2018). This work implied that subtle changes in ion channel expression can cause changes to cell physiology and that multiple combinations of ion channel densities may give similar firing patterns. This study, taken in combination with our findings in SGNs, suggests that while specific *selector* molecules may establish subtypes (IA, IB, IC), each subtype may represent a wide variety of excitation potentials. The heterogeneity in the expression of ion channels was also observed in the ganglion retinal cells and has been suggested to allow neuron populations to encode more information (Berry et al., 2018). The heterogeneity we observe in these ion channels may be due to cells originating in different tonotopic regions of the cochlea.

Among the three subtypes, the IA cells drew our attention the most, due to their expression of *Lmx1a*. The *Lmx1* family of genes is known to act as selector molecules in a variety of different developmental contexts, including the development of the CNS (Chizhikov et al., 2010; Kee et al., 2017). *Lmx1a* is one of the early determinants of the fate of midbrain dopaminergic (mDA) neuronal generation (Chizhikov et al., 2010; Deng et al., 2011; Yan et al., 2011). Also, to its role development, it was recently shown to play an important role in maintaining adult mDA circuitry (Doucet-Beaupré et al., 2015). *Lmx1a* expression has long been seen in the mammalian inner ear and cochlea. Data from human studies reported that loss of function mutations lead to deafness in human populations (Schrauwen et al., 2018; Wesdorp et al., 2018). Furthermore, mutations in *Lmx1a* in mice lead to the improper establishment of the sensory-non-sensory regions of the ear, leading to altered ear morphology (Koo et al., 2009). *Lmx1a* has been suggested to be part of the transcription factor network, together with *Gata3*, that define SGN from the VGN population in early development stages (Appler and Goodrich, 2011). Besides, loss of *Lmx1a* also leads to an expansion of the vestibular ganglion region of the inner ear (Huang et al., 2018). Despite these reports, which establish the importance of *Lmx1a* in the inner ear, little attention has been paid to the expression of *Lmx1a* in the SGN population. Our single-cell high-throughput qPCR reveals a population of *Lmx1a* positive cells at P3, whose abundance decreased with age toward P12. We further validated the selective expression of *Lmx1a* in Type IA SGNs by generating a triple transgenic mouse model using *Lxm1a*-Cre X tdTomato and *Prph*-GFP. We observed that the subset of Type I SGNs was labeled in red, but not the Type II *Prph*-GFP.

To fully establish the *bona fide* role of IA neurons, future studies will have to assess the electrophysiological properties of these *Lmx1a* positive cells. From their expression of *Snap25* and *Grm5*, we hypothesize that IA cells will represent neurons with a high spontaneous firing rate property. However, it is also possible that *Lmx1a* will not define a physiologically homogeneous subpopulation (i.e., only high or low threshold cells), as we observe that they have varied expression patterns of effectors genes such as *Scn1a, Scn9a, Scn1b, and Grm5*. These heterogeneous features will be better explored by deep-sequencing studies that can assess the full transcriptome of each IA SGN, and by electrophysiology experiments using specific blocking agents against each channel.

Taken together, our data provide several novel markers of Type I cells (*Zic1, Pax6*, and *Nfix)* and their subtypes (*Lmx1a, Mfap4, Fzd2, Slc4a4, Cacna1a*) and will enable future efforts to generate subtype-specific reporter lines. These tools will allow the study of the precise interplay of intrinsic genetic factors and extrinsic cues in defining SGNI diversity and their differential susceptibility to noise-induced hearing impairment.

## Data Availability Statement

All datasets generated for this study are included in the article/Supplementary Material.

## Ethics Statement

The animal study was reviewed and approved by Stanford University Institutional Animal Care and Use Committee.

## Author Contributions

FG analyzed and visualized the data and wrote the manuscript. LDT collected the data. MM conceptualized and oversaw the study and data analysis and wrote the manuscript.

## Conflict of Interest

The authors declare that the research was conducted in the absence of any commercial or financial relationships that could be construed as a potential conflict of interest.

## Supplementary Material

FIGURE S1 **(A)** P8 cultured SGN neurons immunostained for TUJ1, in red. **(B)** P8 cultured SGN neurons stained with DAPI, in blue. **(C)** Quantification of total cultured cells after dissection as determined by DAPI and neurons determined by TUJ1 with and without Trypsin (*n* = 3 independent neuronal cultures). **(D–H)** Representative FACS gating strategy for separation of type I spiral ganglion neurons. Percentage (%) of cells in each gate **(D–H)** are shown in red. Cell debris was removed by plotting FSC and SSC to remove doublets and tissue clumps **(D–F)**. Dead cells were removed by staining cells with Sytox Red **(G)**. Finally, we gated tdTomato and GFP cells **(H)** and collected high tdTomato positive cells (outlined in the box).

FIGURE S2 Spiral ganglion neurons single-cell multiplex qRT-PCR quality control. **(A)** *B-actin* **(B)** *Gapdh*, **(C)** percentage of genes expressed per cell. **(D)** Summary of P8 healthy cells, negative control, death, and doublet cells. **(E)** Cells were excluded based on *B-actin* and *Gapdh* expression, lower Log2Ex values indicate death cells or empty wells, and higher Log2Ex values indicate doublet. **(F)** Healthy cells expressed 52.2 ± 13.6% genes, death cells expressed 13.8 ± 4.3% genes, and doublet cells expressed 85.93 ± 0.7% genes of 96 genes analyzed in 96 cells.

FIGURE S3 **(A)** UMAP projection of SGN cells, colored by the FACs gating, green for GFP-Prph, red for tdTomato. **(B)** UMAP projection of SGN cells at P8. Each cell is colored by the expression of genes enriched in Type I cells: *Cadps2*, *Tmem178*, and *Cacna1a*. The expression scale is given on the right. **(C–F)** UMAP projection of SGN cells at P8. Each cell is colored by the expression of **(C)** *Bactin*, **(D)** *Map2*, **(E)** *Gapdh*, and **(F)** *Tubb3*. The expression scale is given on the right. **(G)** UMAP projection of the Type SGNs at postnatal day 8. Each point represents a cell. Cells clustering to Type IA, IB, IC are colored in purple, blue, and red, respectively. Type II cells are colored in brown. The major gene markers for each subtype are given on the right. Two additional clusters were found (in yellow and green) but are not considered in this analysis as they did not have clear marker genes. Cells in gray were not clustered into any of the subgroups. Partially reproduced from Figure 3A for clarity. **(H)** Probability calculated by HDBSCAN that each cell belongs to the cluster assigned in **(G)**. The color key is given to the right. **(I)** Clustering tree generated by the HDBSCAN algorithm to generate clusters in **(G)**.

FIGURE S4 **(A)** Principal components were analyzed, and PC1 to PC10 were plotted in X-axis with the respective variance in Y-axis. **(B)** K-means clustering was performed. The stable numbers of clusters were chosen based on Gap statistic. The dotted line indicates three stable clusters. **(C)** K-means clustering of PC1 and PC2 with pre-defined three clusters (*K* = 3). Black, red, and green dots represent cluster-1, cluster-2, and cluster-3 respectively. PC1 and PC2 are plotted in X-axis and Y-axis, respectively. **(D)** Cluster-1 specific genes are *Cacna1a*, *Lmx1a*, *Nrp1*, *Snap25* and *Timp2*. The cluster-2 specific genes are *Gria3*, *Sclc4a4*, *Cacna1a*. The cluster-3 specific genes are *Fzd2* and *Mfap4*. **(E–J)** representative genes in each cluster were plotted in PC1 and PC2. A color key (horizontal bar) with red, green, and gray colors represents high, low, and not detectable of genes expression respectively in cells.

FIGURE S5 Hierarchical clustering of the same cells analyzed for Kmeans clustering. Three clusters labeled in black, red, and green.

FIGURE S6 **(A–C)** Heat maps of gene expression in Type II, Type IA, Type IB, and Type IC Genes are grouped by transcription factors, signaling, physiology, and guidance and adhesion and **(A)** P3, **(B)** P8, **(C)** P12. Each square represents the average expression in each cell category. **(D–E)** Representative images of *in situ* hybridizations of *Cacna1a* at **(D)** P3 and **(E)** P8 in the cryopreserved whole cochlea. **(F)** Representative images of *in situ* hybridization for *Nefm* at P8 as a positive control.

FIGURE S7 **(A)** UMAP projection of SGN cells at P3 from Peptitpre et al. Each point represents a cell, which is colored by the gene count of *Calb1*. The scale is given at the right. **(B)** UMAP projection of SGN cells at P3 from this study colored by expression of *Calb1*. **(C)** Violin plots of the expression level of *Calb1* at P3, P8, and P12. The different subtypes are colored and indicated on the top. **(D–E)** Data presented as in **(A)** for *Zic1* and *Zic5*. **(F)** Microarray values for *Zic1* and *Zic5* at P0 and P6 in bulk SGN samples taken from Lu et al. (2011). **(G–K)** Data presented as in **(A)** for *Lmx1a, Mfap4, Fzd2, Slc4a4* and *Cacna1a*.

TABLE S1 Raw data + clustering.
