# Corrigendum: Single-Cell RNA Analysis of Type I Spiral Ganglion Neurons Reveals a *Lmx1a* Population in the Cochlea

**DOI:** 10.3389/fnmol.2021.686790

**Published:** 2021-05-05

**Authors:** Fiorella Carla Grandi, Lara De Tomasi, Mirna Mustapha

**Affiliations:** ^1^Cancer Biology Program, Stanford University, Stanford, CA, United States; ^2^Department of Biomedical Science, University of Sheffield, Sheffield, United Kingdom; ^3^Department of Otolaryngology-Head and Neck Surgery, Stanford University School of Medicine, Stanford, CA, United States

**Keywords:** type I spiral ganglion neurons, single-cell transcriptome, *Lmx1a*, development, cochlea

In the original article, there was an error. Dr. Gopal Pramanik was acknowledged without his agreement. As per his request, we are removing his name and contribution from the acknowledgment paragraph.

A correction has been made to the *Acknowledgments*. The corrected paragraph is shown below.

“We sincerely thank Drs. Stefan Heller and Marta Milo for help with data analysis, and Dr. Walter Marcotti for helpful discussion. We also thank Dr. Kathleen J. Millen for generously providing the Lmx1a-cre mice and Dr. Theresa Zwingman for answering the many questions we had concerning these mice (Seattle Children's Research Institute Center for Integrative Brain Research, The University of Washington). We thank Dr. Joseph P. Sarsero (Murdoch Children's Research Institute, Australia) for sharing Peripherin-GFP mice with us and Dr. Lin Gan (Flaum Eye Institute, University of Rochester School of Medicine) for sharing Bhlhb5-cre line.”

The authors apologize for this error and state that this does not change the scientific conclusions of the article in any way. The original article has been updated.

