# Supplementary material for: Single-Cell RNA Analysis of Type I Spiral Ganglion Neurons Reveals a Lmx1a Population in the Cochlea
Source: Front Mol Neurosci. 2020 May 25;13:83. doi: 10.3389/fnmol.2020.00083 (PMC7261882; doi:10.3389/fnmol.2020.00083)

Supplementary 1 (related to Fig. 1)

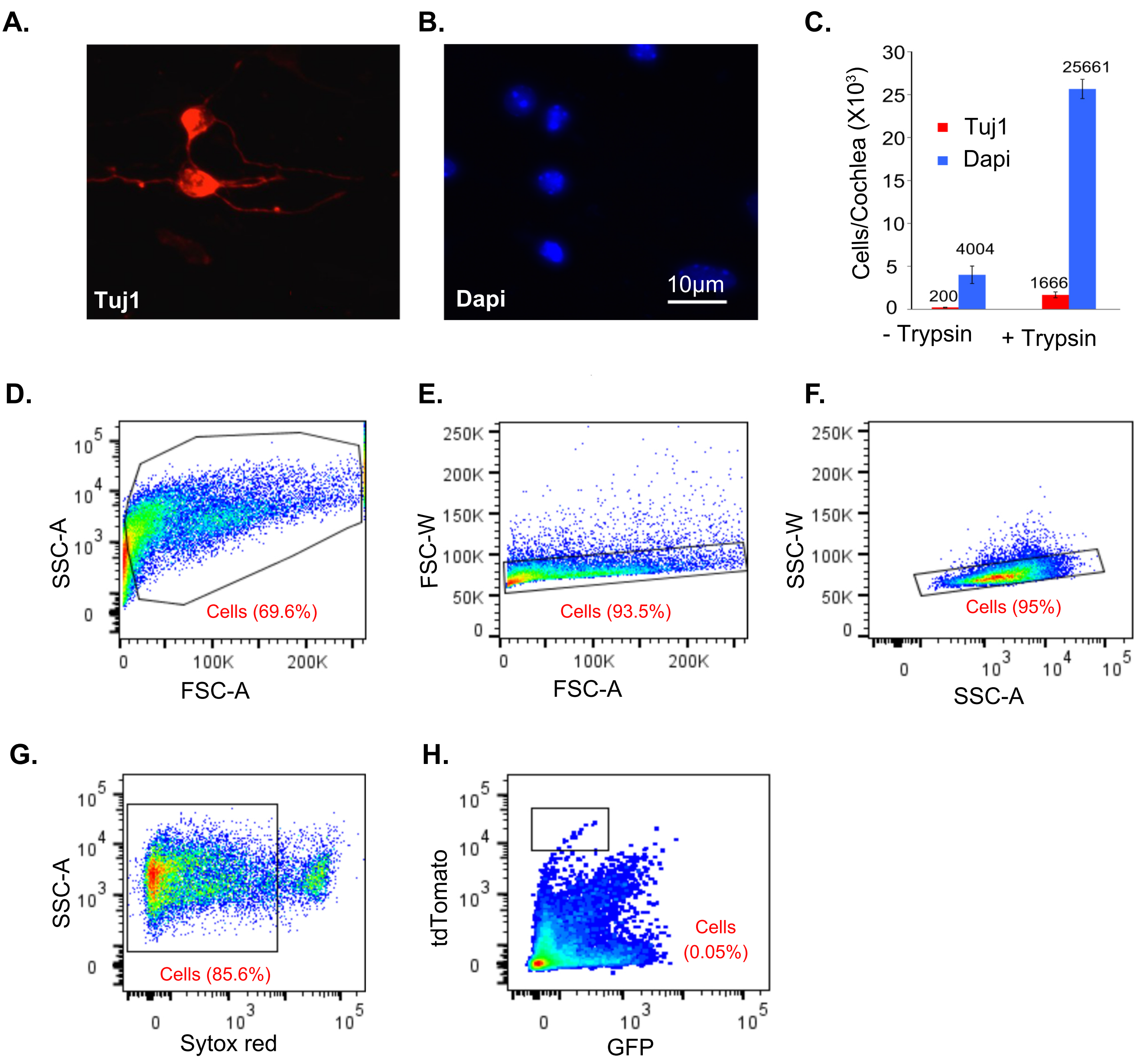

Supplementary Figure 2 (related to Figure 2)

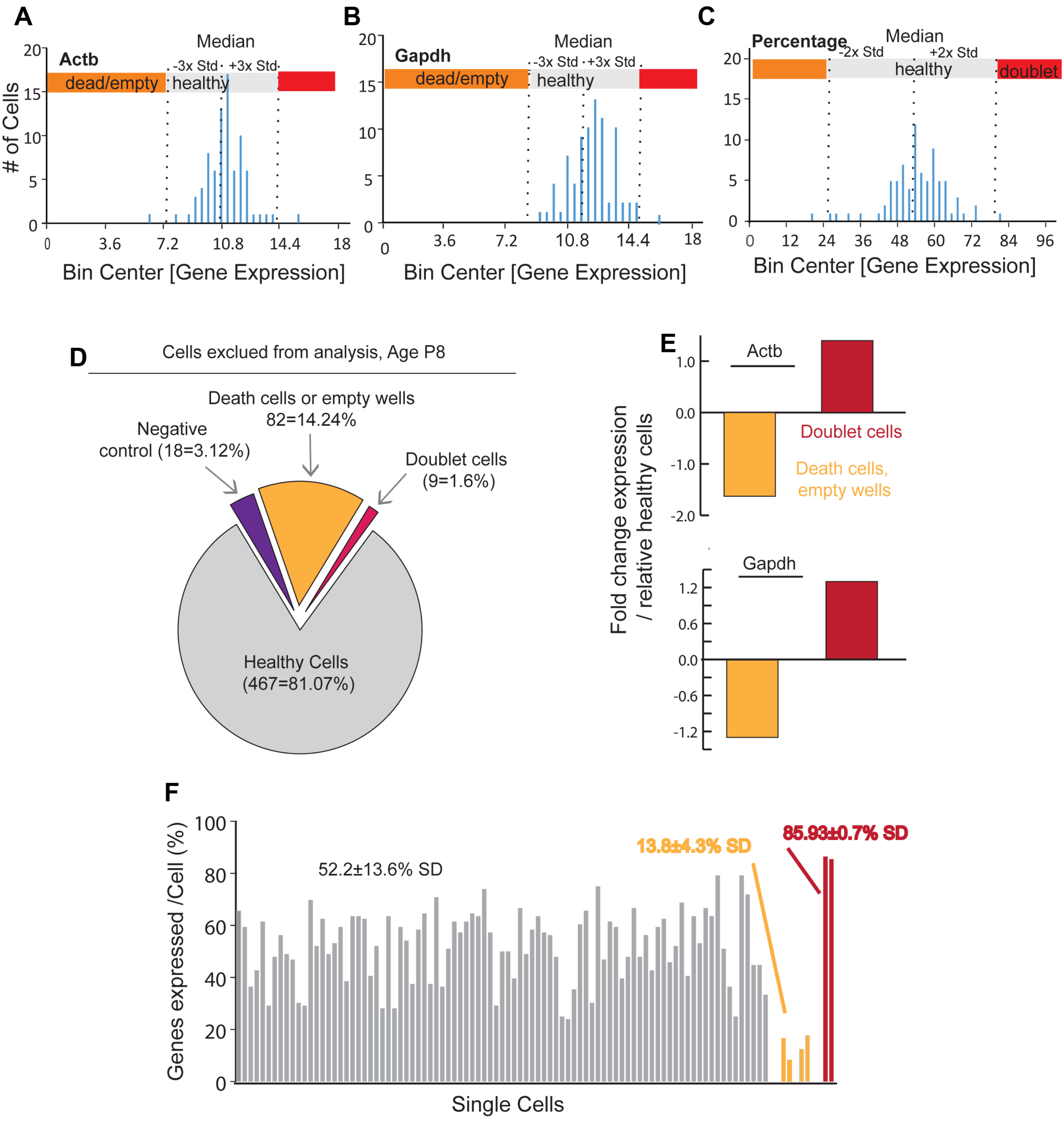

Supplementary Figure 3 (related to Figure 3&4)

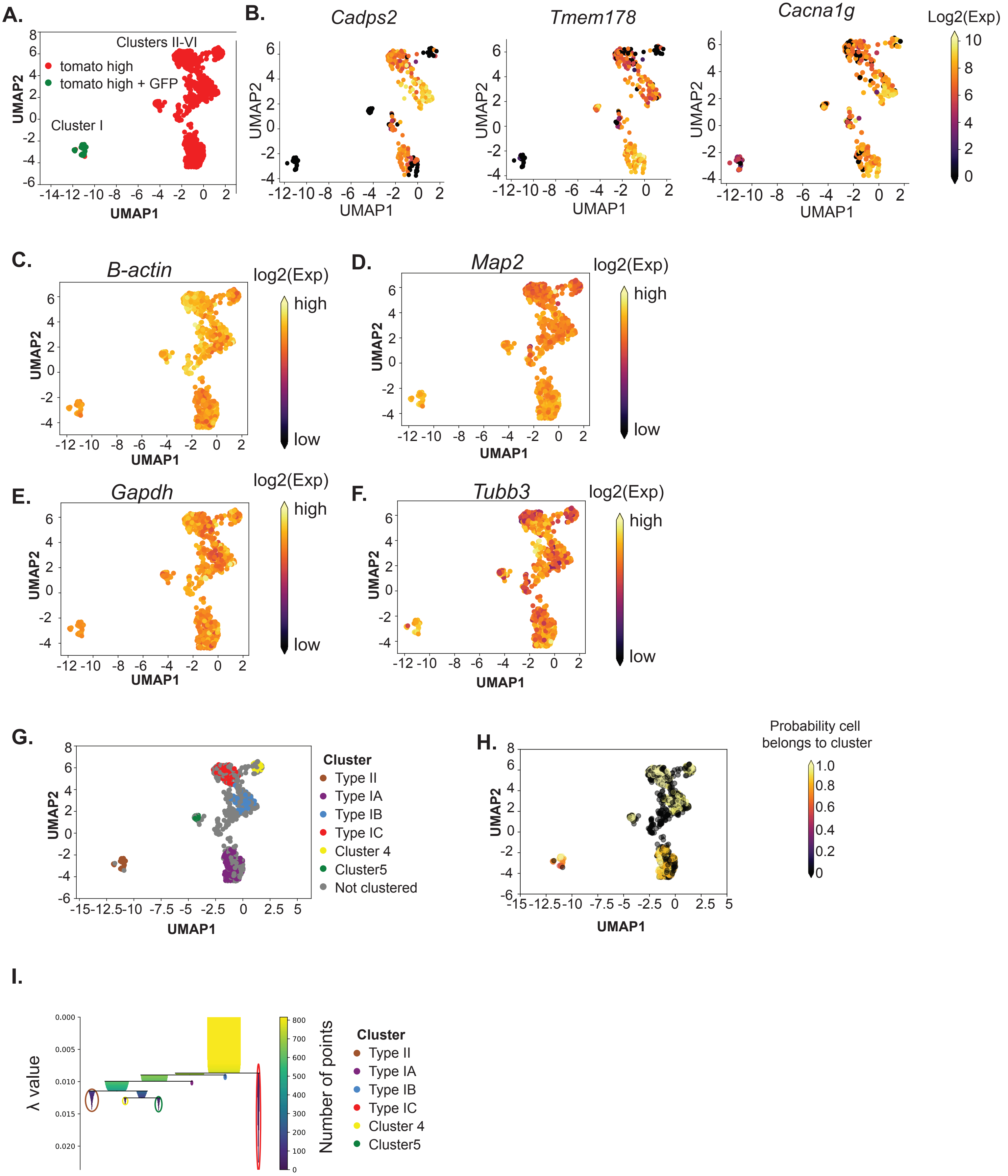

Supplementary Figure 4 (related to Figure 3)

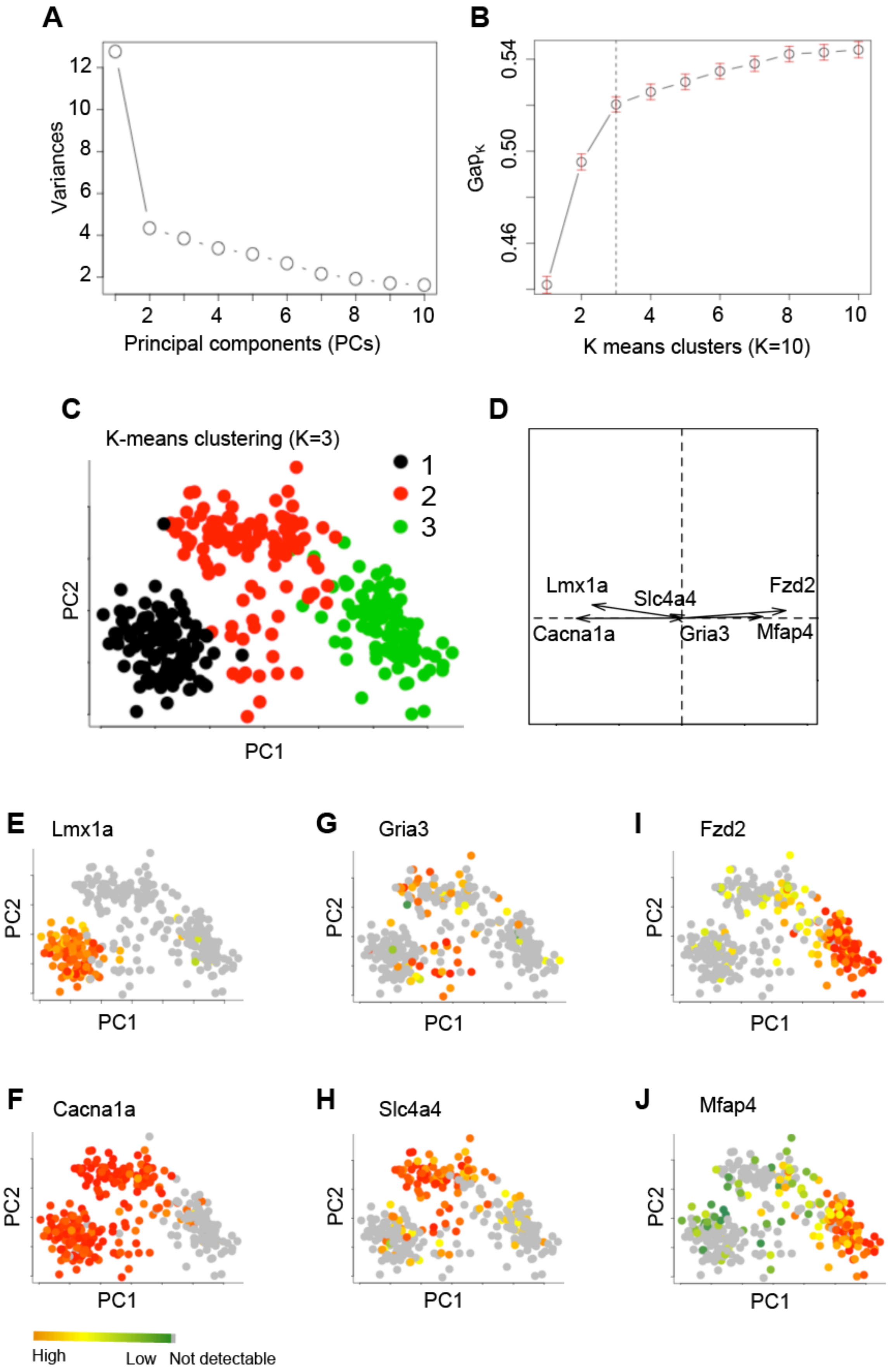

Supplementary Figure 5 (related to Figure 3)

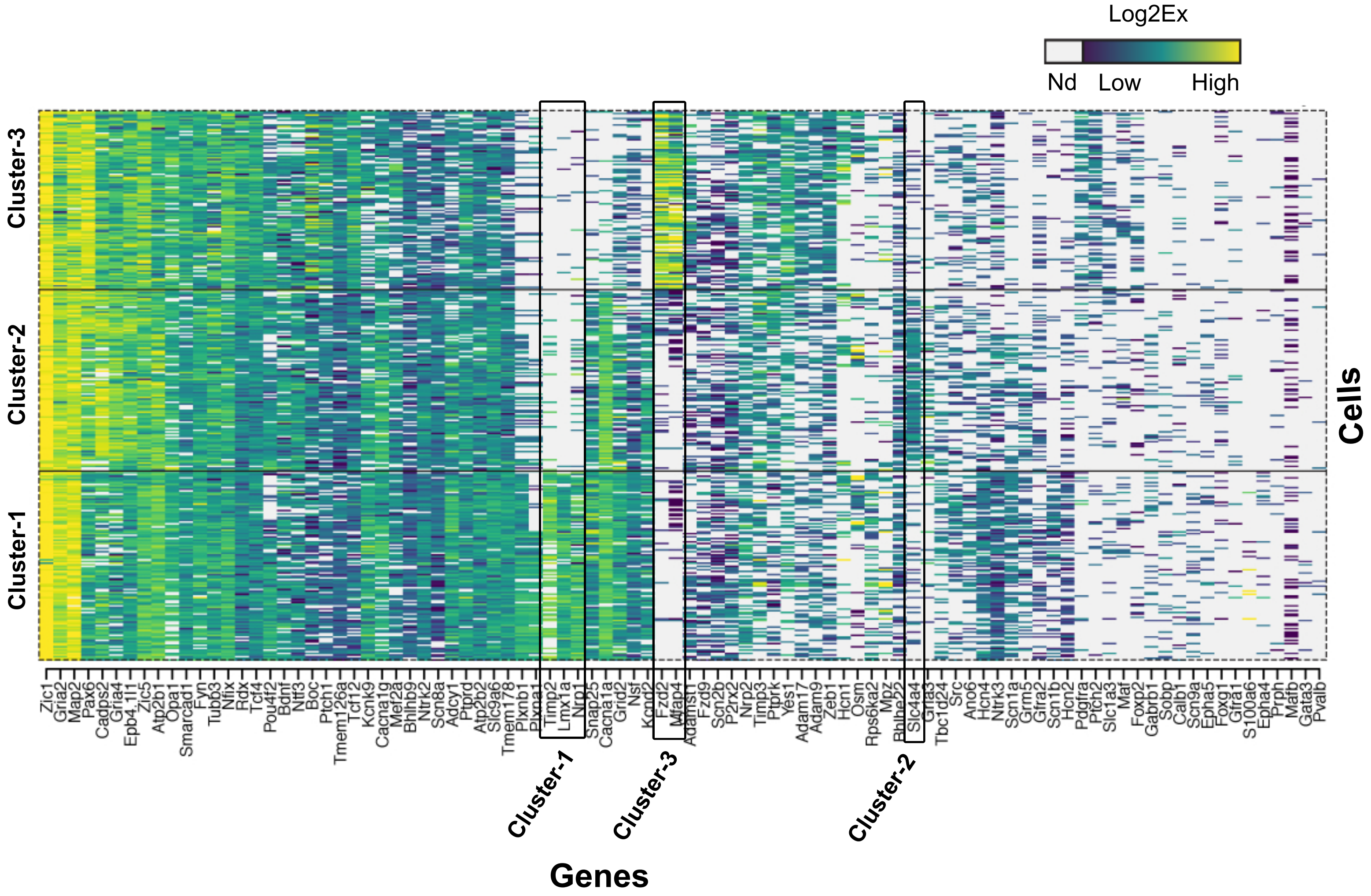

Supplementary Figure 6 (related to Figure 5&6)

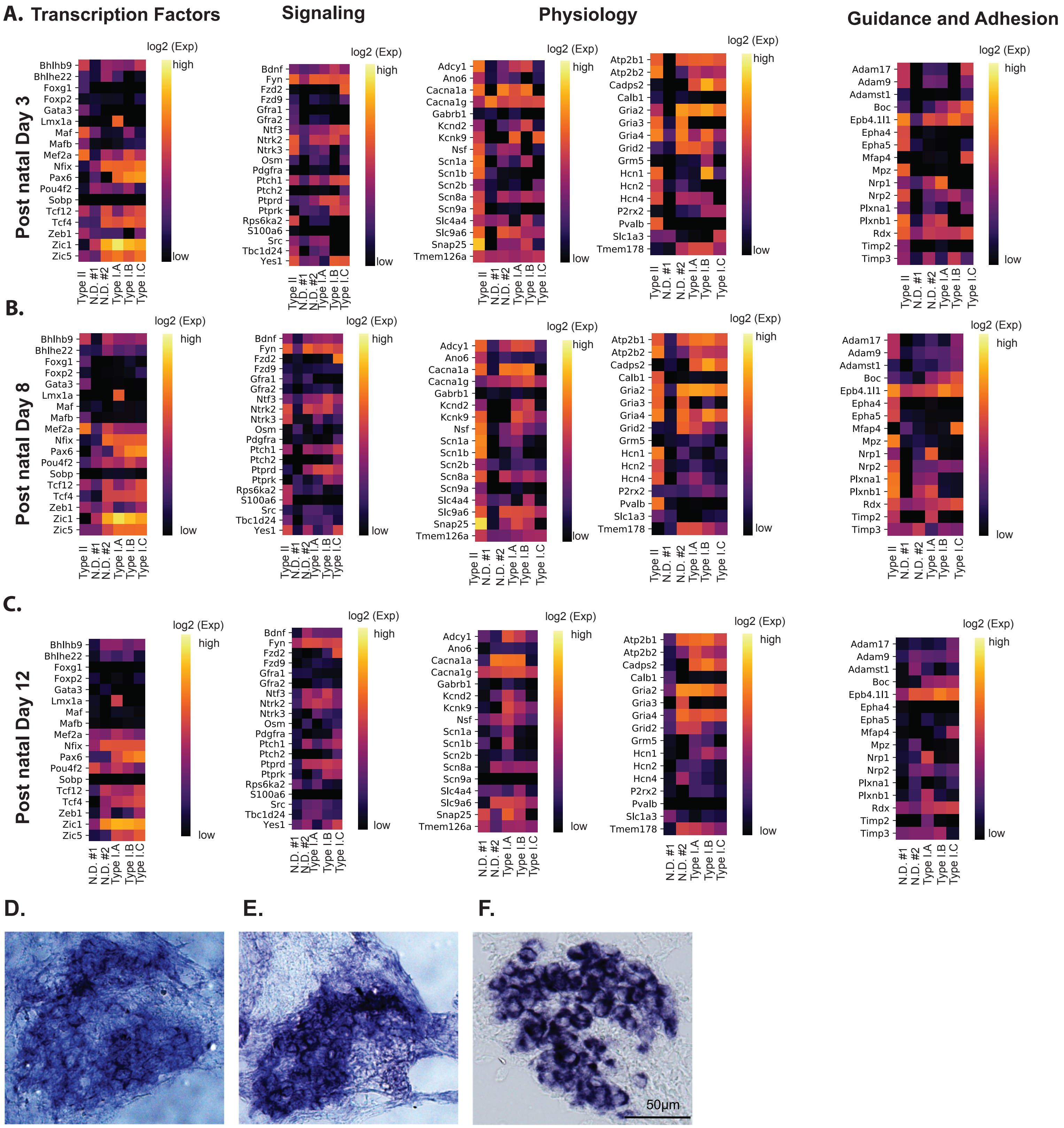

P3 *Cacna1a*   P8 *Cacna1a*   P8 *Nefm*

Supplemental Figure 7

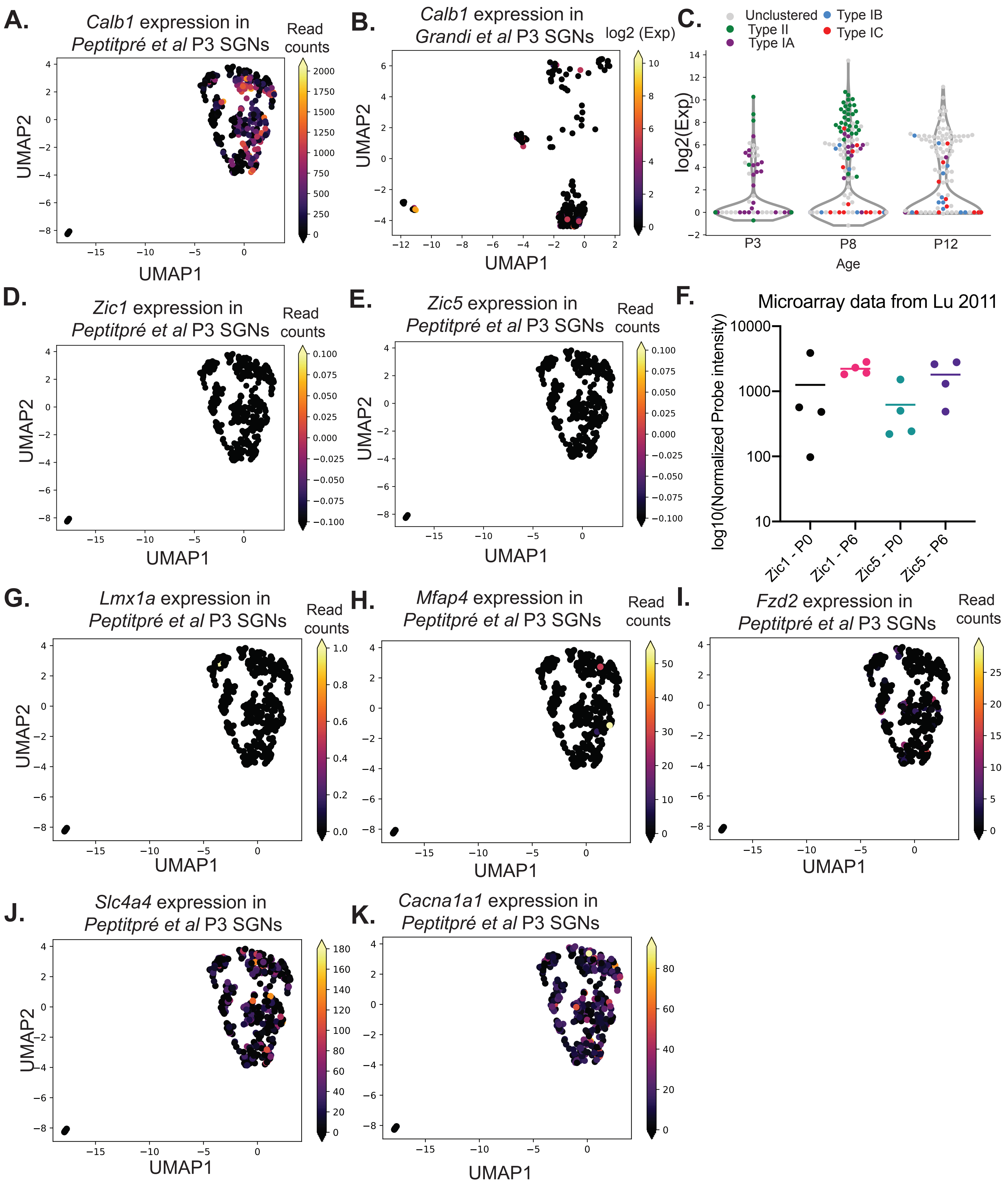

Supplement: FIGURE S1 — (A) P8 cultured SGN neurons immunostained for TUJ1, in red. (B) P8 cultured SGN neurons stained with DAPI, in blue. (C) Quantification of total cultured cells after dissection as determined by DAPI and neurons determined by TUJ1 with and without Trypsin (n = 3 independent neuronal cultures). (D–H) Representative FACS gating strategy for separation of type I spiral ganglion neurons. Percentage (%) of cells in each gate (D–H) are shown in red. Cell debris was removed by plotting FSC and SSC to remove doublets and tissue clumps (D–F). Dead cells were removed by staining cells with Sytox Red (G). Finally, we gated tdTomato and GFP cells (H) and collected high tdTomato positive cells (outlined in the box). [file Data_Sheet_1.pdf]
